# A Treatment Plant Receiving Waste Water from Multiple Bulk Drug Manufacturers Is a Reservoir for Highly Multi-Drug Resistant Integron-Bearing Bacteria

**DOI:** 10.1371/journal.pone.0077310

**Published:** 2013-10-29

**Authors:** Nachiket P. Marathe, Viduthalai R. Regina, Sandeep A. Walujkar, Shakti Singh Charan, Edward R. B. Moore, D. G. Joakim Larsson, Yogesh S. Shouche

**Affiliations:** 1 Microbial Culture Collection (MCC), National Center for Cell Science, Pune, Maharashtra, India; 2 Culture Collection University of Gothenburg (CCUG), Gothenburg, Sweden; 3 Department of Infectious Diseases, Institute of Biomedicine, The Sahlgrenska Academy at the University of Gothenburg, Gothenburg, Sweden; National University of Singapore, Singapore

## Abstract

The arenas and detailed mechanisms for transfer of antibiotic resistance genes between environmental bacteria and pathogens are largely unclear. Selection pressures from antibiotics in situations where environmental bacteria and human pathogens meet are expected to increase the risks for such gene transfer events. We hypothesize that waste-water treatment plants (WWTPs) serving antibiotic manufacturing industries may provide such spawning grounds, given the high bacterial densities present there together with exceptionally strong and persistent selection pressures from the antibiotic-contaminated waste. Previous analyses of effluent from an Indian industrial WWTP that processes waste from bulk drug production revealed the presence of a range of drugs, including broad spectrum antibiotics at extremely high concentrations (mg/L range). In this study, we have characterized the antibiotic resistance profiles of 93 bacterial strains sampled at different stages of the treatment process from the WWTP against 39 antibiotics belonging to 12 different classes. A large majority (86%) of the strains were resistant to 20 or more antibiotics. Although there were no classically-recognized human pathogens among the 93 isolated strains, opportunistic pathogens such as *Ochrobactrum intermedium, Providencia rettgeri,* vancomycin resistant *Enterococci* (VRE), *Aerococcus sp*. and *Citrobacter freundii* were found to be highly resistant. One of the *O. intermedium* strains (ER1) was resistant to 36 antibiotics, while *P. rettgeri* (OSR3) was resistant to 35 antibiotics. Class 1 and 2 integrons were detected in 74/93 (80%) strains each, and 88/93 (95%) strains harbored at least one type of integron. The qPCR analysis of community DNA also showed an unprecedented high prevalence of integrons, suggesting that the bacteria living under such high selective pressure have an appreciable potential for genetic exchange of resistance genes via mobile gene cassettes. The present study provides insight into the mechanisms behind and the extent of multi-drug resistance among bacteria living under an extreme antibiotic selection pressure.

## Introduction

The emergence of multi-drug resistance in bacterial human pathogens is one of the most serious challenges for health care globally. Pathogens that earlier were sensitive to antibiotics are becoming resistant by mutations in their preexisting DNA or by acquisition of DNA containing resistance genes [1]. Most of the antibiotic resistance genes carried by pathogens have their origins in environments other than the clinical world and the normal bacterial flora of disparate environments are thought to be the reservoirs of these resistance genes [2]–[7]. The ability of bacteria to exchange genes across species boundaries is an important factor in the spread of acquired antibiotic resistance. This often is facilitated by mobile genetic elements such as plasmids, transposons, integrons and genomic islands that harbor antibiotic resistance genes.

Recent studies have shown that antibiotic resistance genes are ancient and were present in the environment long before the antibiotic era [8], [9]. The overuse and misuse of antibiotics has led to increased selection pressures, also in the environment. In bacterial communities exposed to sufficient selection pressure from exposure to antibiotics, resistance genes may increase radically in abundance within the populations [10]–[12]. Such increases may also be accompanied by increased frequencies in genetic elements facilitating their mobility [12], [13]. Thus, exposure to antibiotics is expected to increase the risk for the transfer of resistance between bacterial species. This is true not only during antibiotic treatment of humans and animals, but also when a sufficiently high level of antibiotic contamination reaches the external environment. Environments exposed to hospital and agricultural waste typically contain both antibiotic resistant bacteria as well as moderately elevated levels of antibiotics, providing examples of ecosystems wherein antibiotics may exhibit the potential for selecting resistant strains [14], [15]. Recently, wastewaters from antibiotic manufacturing in India, China, Korea and Croatia, as well as environments contaminated by such waste, have been reported to contain excessively high antibiotic concentrations [12], [16]–[22]. We have studied a waste-water treatment plant (WWTP) in Patancheru, near Hyderabad, India operated by Patancheru Environment Technology Limited (PETL) [12], [16], [17], [23]–[27]. The plant receives effluent from approximately 90 regional bulk drug manufacturers and the treated effluent contains a range of drugs including fluoroquinolone antibiotics, such as enrofloxacin, ofloxacin, norfloxacin, lomefloxacin and ciprofloxacin, at concentrations as high as 30 mg/L [16]. The levels of ciprofloxacin are even higher than those attained in the blood of patients taking this broad-spectrum antibiotic. Accordingly, concerns have been expressed that antibiotic resistance will be promoted in such processes [16], [28].

A recent study, applying next-generation sequencing of microbial community DNA, demonstrated the promotion of resistance genes to several classes of antibiotics in river sediment contaminated by effluent from the PETL WWTP [12]. Additionally, elements associated with genetic mobility were detected in significantly higher frequencies down-stream from the treatment plant. Cultivation-independent approaches for analyzing microbial communities, such as metagenomic DNA sequencing, have apparent advantages compared to cultivation-dependent methods, particularly when studying environmental bacterial communities where a significant proportion of community members are “uncultivable”. However, the cultivation-independent strategies may be unable to provide information regarding the resistance pattern of a particular species, whether the same strains are resistant to multiple classes of antibiotics or whether different strains belonging to same species have different drug resistance pattern. Furthermore, cultivation-independent approaches do not confirm the phenotypic expression of resistance in strains.

We hypothesize that activated sludge treatment in the presence of multiple antibiotics would result in sludge and wastewater dominated by multi-drug resistant bacteria. To investigate this, we used a cultivation-dependent approach. Bacterial strains (n = 93) from within the PETL WWTP were isolated and their resistances against 39 antibiotics belonging to 12 different classes were assessed. The presence of class 1 and class 2 integrons among the isolated strains was investigated by PCR and quantitation of the integrons in total community DNA was performed by qPCR, in order to estimate horizontal gene transfer (HGT) potential of the bacteria.

## Materials and Methods

### Sample collection

Batch samples were collected from the WWTP operated by PETL in the month of March, 2007. The samples were collected with appropriate permissions from PETL authorities. Water samples from the equilibrator (EQR), aeration tank no.1 (AER1), aeration tank no. 2 (AER2), settling tank (STL) and sludge samples from the secondary sludge (SS), dewatered sludge (DS) and the old dried sludge (OS) were collected in sterile containers, stored on ice and transported to our laboratory in Pune, Maharashtra, India.

### Isolation of bacteria

Bacterial strains were isolated by the spread plate method. Briefly, 1.0 ml of the liquid samples or 1.0 g (wet weight) of the sludge samples were suspended in 9.0 ml of sterile phosphate-buffered saline (PBS), pH 7.2. The serial dilutions were plated onto Luria-Bertani (LB) agar, Tryptic Soy agar (TSA) and R2A agar media and incubated at 30°C, for 24 to 48 hours. Isolated colonies of different morphologies were selected and re-streaked on the respective media to obtain pure cultures. The strains were stored as glycerol stocks at −70°C.

### DNA extraction, PCR, Cloning and Sequencing

Genomic DNA was extracted from freshly grown bacterial strains using a standard phenol/chloroform method [29]. The total community DNA from concentrated water (100 ml) and sludge (0.25 g) samples were extracted using the PowerSoil™ DNA Isolation Kit (MoBio Laboratories Inc., Carlsbad, USA). The concentrations of all DNA samples were adjusted to 20 ng/µl for further experiments.

The strains were identified by 16S rRNA gene sequencing, using primers 27F and 1488R as described earlier [30]. The presence of integrons in the strains was investigated by PCR using primers described previously [31]. The list of primers used in the study is represented in Table 1. PCR-amplification products were purified and sequenced using the BigDye Terminator Cycle Sequencing Ready Reaction Kit v 3.1 and an automated 3730xl DNA analyzer (Applied Biosystems Inc., USA).

**Table 1:** PCR-primers used in the present study.

Primer Name	Sequence 5′–3′	Description and Reference
27F	CCAGAGTTTGATCMTGGCTCAG	16S rRNA gene forward/qPCR forward [30]
1488R	CGGTTACCTTGTTACGACTTCACC	16S rRNA gene reverse [30]
343R	GACTACCAGGGTATCTAATCCTGTT	qPCR 16S rRNA gene reverse [30]
Int1RTF	CCTCCCGCACGATGATC	qPCR integrase 1 forward [36]
Int1RTR	TCCACGCATCGTCAGGC	qPCR integrase 1 reverse [36]
Intl2RTF	TTATTGCTGGGATTAGGC	qPCR integrase 2 forward [36]
Int2RTR	ACGGCTACCCTCTGTTATC	qPCR integrase 2 reverse [36]
Int1F	GGGTCAAGGATCTGGATTTCG	PCR integrase1 forward [31]
Int1R	ACATGGGTGTAAATCATCGTC	PCR integrase 1 reverse [31]
Int2F	CACGGATATGCGACAAAAAGGT	PCR integrase 2 forward [31]
Int2R	GTAGCAAACGAGTGACGAAATG	PCR integrase 2 reverse [31]
integ-1	GGCATCCAAGCAAG	Class 1 integron variable region [32]
integ-2	AAGCAGACTTGACCTGA	Class 1 integron variable region [32]
hep51	GATGCCATCGCAAGTACGAG	Class 2 integron variable region [33]
hep74	CGGGATCCCGGACGGCATGCACGATTTGTA	Class 2 integron variable region [33]

Integron gene cassettes from the pooled community DNA from all sites were amplified, using primers previously described [32], [33]. The total community DNA was used for amplifying integron gene cassettes associated with the total bacterial population in a given sample. PCR-amplification products were separated by agarose gel electrophoresis; bands of 1.0 kb and 1.5 kb for class 1 and 1.0 kb for class 2 integron gene cassettes respectively, were eluted from the gels using the Gene Elute Gel Extraction Kit (Sigma-aldrich, St Louis, USA). The eluted products were cloned into the pGEM-T vector (Promega, Madison, USA) and transformed into *Escherichia coli* JM109 High Efficiency Chemical Competent cells (Promega, Madison, USA). The clones with inserts were detected by PCR and the inserts were sequenced. The sequences were assembled using Chromas Pro software and identifications of cassettes were done using BLASTX (http://blast.ncbi.nlm.nih.gov/Blast.cgi).

### Antibiotic resistance profile determinations

The antibiotic sensitivity profiles of the strains were characterized using the disc diffusion method [34]. Thirty-nine antibiotics, representing 12 classes, were used to characterize the strains as resistant, sensitive or intermediately resistant, according to the standards of the Clinical and Laboratory Standards Institute [35]. The quantity of antibiotic per disc and the cut-off value of the respective zones of inhibition used for the study are reported in Table S1.

### Quantification of 16S rRNA gene, class 1 and class 2 integrons

Integrons were quantified by real-time quantitative PCR (qPCR) as described previously [36]. The qPCR for 16S rRNA gene was carried out as described by Marathe *et al.,* [30]. All qPCR reactions were done in triplicate, in a final volume of 25.0 µl, using 1× Power SYBR Green PCR master mix and performed in the ABI 7300 Real-Time PCR System (Applied Biosystems, Inc. USA).

Purified *E. coli* K12 genomic DNA was quantified, using a Nanodrop spectrophotometer (J H Bio innovations, Secunderabad, India), and serial dilutions were used for 16S rRNA gene reference preparation. For the preparation of integron qPCR standards, conserved regions (101 bp) of the genes for the class 1 and class 2 integrase were amplified from total community DNA, using the primers presented in Table 1, and purified by the gel elution method. The purified PCR products were cloned into the pGEM-T vector (Promega, Madison, USA) and transformed into *E. coli* JM109 high efficiency chemical competent cells. Plasmids with inserts were extracted from the clones using Plasmid Miniprep Kit (Sigma-aldrich, St Louis, USA), quantified using a Nanodrop spectrophotometer (J H Bio innovations, Secunderabad, India) and the serial dilutions of these plasmids were used as standards. The mass of a single *E. coli* genome or plasmid containing insert was calculated by multiplying the number of base pairs by their average mass (1.096e-21 g/bp). The copy number was calculated by dividing the amount of standard DNA by the mass of single genome or a single copy of the plasmid. The copy number obtained for 16S rRNA gene was multiplied by a factor of 7, as *E. coli* K12 genome (Genbank accession number NC_000913) contains 7 copies of 16S rRNA gene. Copy numbers in the samples were calculated by comparison with the standard curves for respective genes. The efficiency of the PCR (E) was taken into account by the equation E = 10^−1/slope^ –1.

## Results

### Resistance profiles of the strains

A total of 93 strains of bacteria were isolated and analyzed. The identifications of the strains, based upon comparative 16S rRNA gene sequence analysis, are presented in Table 2. The 16S rRNA gene sequences were submitted to Genbank under accession numbers KC634231–KC634323. The composition of the cultured strains was dominated by *Proteobacteria* (74%) and *Firmicutes* (24%). Among the *Proteobacteria*, the *Gammaproteobacteria* were the most prevalent (56%), followed by the *Alphaproteobacteria* (22%) and the *Betaproteobacteria* (19%). *Actinobacteria* comprised the remaining strains. The *Gammaproteobacteria* is a class containing several clinically important taxa, such as *Enterobacteriaceae*, *Vibrionaceae* and *Pseudomonadaceae*. Some species were isolated from only one or a few sampling sites, *e.g*., *Advenella mimigardefordensis* (A1R-10 and A2T-5) was isolated only from aeration tanks, Table 2, while a few species were isolated from several different sampling sites, *e.g*., *Ochrobactrum intermedium* (SR-2, SSR-6, ER-1, A1R-2, A1T, A2R-4 and A2T-3) and *Pseudomonas caeni* (A2T-6, ET-1, DST-1 and SST-1).

**Table 2:** Identity and antibiotic sensitivity pattern of the bacterial strains isolated from the PETL WWTP against 39 antibiotics.

Strain	Identity	Resistant	Sensitive	Intermediately resistant
A1R-10	*Advenella mimigardefordensis*	15	21	3
A2T-5	*Advenella mimigardefordensis*	29	9	1
SR-9	*Aerococcus urinaeequi*	20	14	5
DSR-6	*Aerococcus urinaeequi*	20	14	5
OST-1	*Aerococcus urinaeequi*	20	17	2
OST-12	*Aerococcus viridans*	20	14	5
ST-1	*Alcaligenes faecalis*	33	6	0
ST-2	*Alcaligenes faecalis*	18	17	4
OST-2	*Alcaligenes faecalis*	19	15	5
OST-9	*Alcaligenes faecalis*	22	10	7
OST-14	*Alcaligenes faecalis*	22	10	7
ET-7	*Alcaligenes sp.*	17	17	5
A2T-8	*Aquamicrobium defluvii*	18	14	7
A1T-8	*Bacillus cereus*	19	8	12
A2T-4	*Bacillus cereus*	27	9	3
ER-5	*Bacillus cereus*	19	8	12
ET-4	*Bacillus cereus*	20	11	8
DSR-1	*Bacillus cereus*	4	28	7
DSR-2	*Bacillus cereus*	17	14	8
A1R-4	*Bacillus flexus*	3	32	4
A2R-5	*Bacillus safensis*	10	16	13
A2R-6	*Bacillus subtilis*	5	28	6
OSR-1	*Bacillus subtilis*	4	32	3
SSR-1	*Bacillus subtilis*	6	26	7
SSR-2	*Bacillus subtilis*	6	26	7
SSR-3	*Bacillus thuringiensis*	18	18	3
SR-6	*Bordetella trematum*	15	23	1
SR-13	*Bordetella trematum*	14	22	3
OST-3	*Brevibacterium samyangense*	9	24	6
DST-6	*Brevundimonas diminuta*	25	9	5
ST-7	*Brevundimonas naejangsanensis*	19	18	2
ST-6	*Castellaniella denitrificans*	22	15	2
ST-8	*Castellaniella denitrificans*	15	22	2
SR-5	*Citrobacter freundii*	21	15	3
OST-4	*Corynebacterium sp.*	8	28	3
SR-3	*Enterobacter hormaechei*	26	11	2
A1T-7	*Enterococcus casseliflavus*	26	8	5
OST-6	*Enterococcus gallinarum*	24	10	5
OST-5	*Enterococcus italicus*	19	15	5
OST-10	*Enterococcus italicus*	19	15	5
SR-12	*Ochrobactrum intermedium*	27	9	3
A1R-2	*Ochrobactrum intermedium*	34	4	1
A1R-7	*Ochrobactrum intermedium*	34	5	0
A1R-9	*Ochrobactrum intermedium*	32	6	1
A1T-4	*Ochrobactrum intermedium*	28	9	2
A1T-5	*Ochrobactrum intermedium*	26	9	4
A2R-4	*Ochrobactrum intermedium*	34	4	1
A2R-8	*Ochrobactrum intermedium*	33	4	2
A2T-3	*Ochrobactrum intermedium*	18	14	7
ER-1	*Ochrobactrum intermedium*	36	1	2
ER-2	*Ochrobactrum intermedium*	28	9	2
SSR-6	*Ochrobactrum intermedium*	22	12	5
SST-7	*Ochrobactrum intermedium*	27	9	3
A1R-6	*Ochrobactrum oryzae*	25	10	4
OST-13	*Paenalcaligenes sp.*	21	13	5
DSR-4	*Providencia rettgeri*	26	8	5
DSR-5	*Providencia rettgeri*	34	3	2
OSR-3	*Providencia rettgeri*	35	3	1
SSR-4	*Providencia rettgeri*	32	4	3
SSR-5	*Providencia rettgeri*	32	3	4
SR-14	*Pseudomonas anguilliseptica*	23	14	2
A2T-6	*Pseudomonas caeni*	28	9	2
ET-1	*Pseudomonas caeni*	19	16	4
ET-5	*Pseudomonas caeni*	31	6	2
ET-6	*Pseudomonas caeni*	22	11	6
DST-1	*Pseudomonas caeni*	29	6	4
DST-2	*Pseudomonas caeni*	22	11	6
DST-3	*Pseudomonas caeni*	22	11	6
DST-4	*Pseudomonas caeni*	29	6	4
DST-5	*Pseudomonas caeni*	27	8	4
DST-7	*Pseudomonas caeni*	22	11	6
DST-8	*Pseudomonas caeni*	29	6	4
SST-1	*Pseudomonas caeni*	29	6	4
SST-2	*Pseudomonas caeni*	15	17	7
SST-3	*Pseudomonas caeni*	29	6	4
SST-4	*Pseudomonas caeni*	29	6	4
SST-5	*Pseudomonas caeni*	29	6	4
SST-6	*Pseudomonas caeni*	29	6	4
SST-8	*Pseudomonas caeni*	29	6	4
SR-11	*Pseudomonas fulva*	29	10	0
ET-2	*Pseudomonas peli*	21	13	5
SR-4	*Pseudomonas plecoglossicida*	30	8	1
SR-7	*Pseudomonas plecoglossicida*	31	7	1
SR-8	*Pseudomonas plecoglossicida*	30	8	1
ST-3	*Pseudomonas sp.*	29	8	2
ST-4	*Pseudomonas sp.*	29	10	0
A1T-2	*Pseudomonas stutzeri*	29	9	1
A1T-3	*Pseudomonas stutzeri*	25	10	4
ER-4	*Pseudomonas stutzeri*	25	10	4
OST-11	*Pseudomonas stutzeri*	21	9	9
OSR-4	*Pseudomonas xanthomarina*	30	6	3
ST-5	*Rheinheimera aquimaris*	23	14	2
SR-1	*Staphylococcus cohnii*	13	21	5

Source of strains: Strains ER1 to ER-5 and ET-1 to ET-7 were isolated from the equilibratior tank; A1R-2 to A1R-10 and A1T2 to A1T8 were isolated from aeration tank No. 1; A2R4 to A2R-8 and A2T-3 to A2T-8 were isolated from aeration tank No. 2; SR-1 to SR-14 and ST-1 to ST-8 were isolated from the settling tank; SSR-1 to SSR-6 and SST-1 to SST-8 were isolated from secondary sludge; DSR-1 to DSR-6 and DST-1 to DST-8 were isolated from dewatered sludge; OSR-1 to OSR-4 and OST-1 to OST-14 were isolated from old dried sludge.

**Figure 1:**
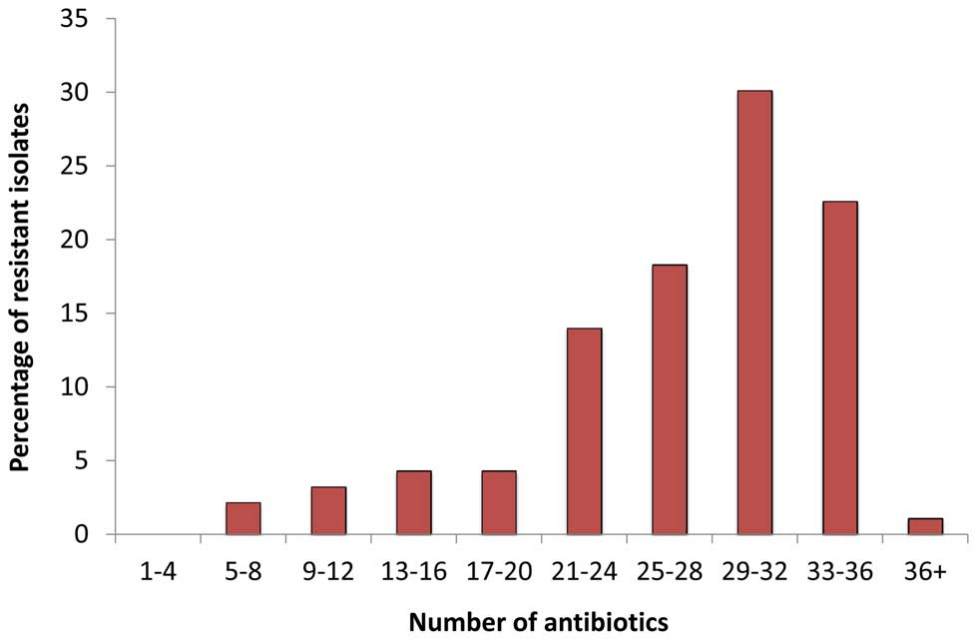
Multi-drug resistance observed among 93 bacterial strains isolated from the PETL WWTP. The X-axis indicates the number of antibiotics (of 39 tested) to which the strains are resistant; the Y-axis indicates the percentage of resistant strains of 93 strains. The resistant and intermediately-resistant phenotypes are grouped together and denoted as resistant.

All strains were resistant to, at least, 5 of the 39 tested antibiotics (Figure 1). A great majority (80/93 = 86%) of the strains were resistant to 20 or more antibiotics (Figure 1), while 53% (49/93) of the strains were resistant to 29 or more antibiotics. The most pronounced resistance was to ampicillin (98%) and mecillinam (98%), two β-lactam antibiotics (Table 3). A majority of strains (62%) were resistant to ciprofloxacin, whereas 20% showed intermediate resistance and 18% were sensitive.

**Table 3:** Distribution of resistance among the strains from PETL WWTP against different antibiotics.

Antibiotic Class	Antibiotic	Resistant strains in %.	Sensitive strains in %.	Intermediately- resistant strains in %.
Aminoglycosides	Gentamicin	9.68	87.10	3.23
	Tobramycin	8.60	76.34	15.05
	Streptomycin	24.73	67.74	7.53
Cephalosporins	Cephalexin	73.12	23.66	3.23
	Cephalothin	74.19	20.43	5.38
	Cefoxitin	68.82	19.35	11.83
	Cefaclor	64.52	32.26	3.23
	Ceftazidime	79.57	12.90	7.53
	Ceftriaxone	45.16	40.86	13.98
	Cefotaxime	51.61	31.18	17.20
Glycopeptides	Teicoplanin	81.72	12.90	5.38
	Vancomycin	82.80	17.20	0.00
Macrolides	Azithromycin	49.46	36.56	13.98
	Erythromycin	67.74	20.43	11.83
Monobactams	Aztreonam	91.40	0.00	8.60
Penicillins	Ampicillin	97.85	2.15	0.00
	Augmentin	58.06	23.66	18.28
	Methicillin	91.40	8.60	0.00
	Penicillin-G	93.55	6.45	0.00
	Piperacillin/Tazobactam	53.76	30.11	16.13
	Ticarcillin	93.55	6.45	0.00
	Mecillinam	97.85	1.08	1.08
Polypeptides	Colistin	40.86	24.73	34.41
Quinolones	Nalidixic acid	75.27	13.98	10.75
	Ciprofloxacin	61.29	18.28	20.43
	Gatifloxacin	41.94	43.01	15.05
	Norfloxacin	54.84	39.78	5.38
	Ofloxacin	39.78	44.09	16.13
	Sparfloxacin	44.09	35.48	20.43
Sulfonamides	Sulfamethoxazole	77.42	6.45	16.13
	Co-Trimoxazole	67.74	30.11	2.15
	Trimethoprim	45.16	33.33	21.51
Tetracyclines	Doxycycline hydrochloride	12.90	80.65	6.45
	Netillin	13.98	84.95	1.08
	Tetracycline	10.75	84.95	4.30
Others	Chloramphenicol	34.41	47.31	18.28
	Fusidic acid	87.10	0.00	12.90
	Nitrofurantoin	62.37	24.73	12.90
	Novobiocin	72.04	15.05	12.90

One of the strains (ER-1 =  CCUG 57381), identified as *Ochrobactrum intermedium*, was resistant to 36 of 39 antibiotics tested, intermediately resistant to 2 antibiotics and was sensitive only to streptomycin. Several other strains of *Ochrobactrum* sp. also exhibited extensive drug resistance, although less than that of strain ER-1 (Table 2). The second most resistant genus was *Providencia.* One of the strains, *P. rettgeri* (OSR-3) was resistant to 35 of the 39 antibiotics tested.

Among strains belonging to the genus *Pseudomonas*, the resistance profiles ranged from resistance against 15 antibiotics, for the strain identified as *P. caeni* (SST-2), to resistance against 31 antibiotics, observed for the strain identified as *P. plecoglossicida* (SR-7). Although strains of *Bacillus* sp. in general, exhibited less extensive resistance profile than many of the other genera analyzed, one of the strains, identified as *B. cereus* (A2T4), was resistant to 27 antibiotics; while one of the *B. cereus* strains (ET-4) and one *B. thuringiensis* (SSR-3) were resistant to 20 and 18 antibiotics respectively. The antibiotic sensitivity data for individual strains is represented in File S1.

The resistance profiles of the strains obtained from different stages of the WWTP treatment process were more or less similar (Table 2). *P. rettgeri* strains from dewatered sludge (DSR-5), old sludge (OSR-3) and secondary sludge (SSR-4) were resistant to 34, 35 and 32 antibiotics respectively. Similarly *Pseudomonas caeni* strains from equilibrator (ET-5), aeration tank (A2T-6), dewatered sludge (DST-4) and secondary sludge (SST-5) were resistant to 31, 28, 29 and 29 antibiotics respectively.

### Integrons and Resistance Gene Cassettes

Class 1 integrons were detected in 74 of 93 strains (80%) and class 2 integrons in 74 strains (80%); eighty eight of 93 strains, (95%) harbored at least one type of integron and both classes of integrons were found in 60 of the strains (65%). The five strains with no integrons were resistant to comparably few antibiotics, *i.e*., 5–8. The list is presented in Table S2. There was no clear difference in the prevalence of class 1 and class 2 integrons between the strains isolated from different stages of WWTP, Table S2.

The gene cassettes associated with the class 1 integrons obtained from pooled total community DNA included: aminoglycoside 6′-n-acetyltransferase (NCBI Accession No. AAM92464); aminoglycoside adenyltransferase (CAQ51305); dihydrofolate reductase type I (AAX46054); haloacid dehalogenase type II (YP_001953944); hypothetical protein Spea_2925 (YP_001502777); OrfC (hypothetical protein) (ABB72863); OXA-2 beta-lactamase precursor (NP_511223); quaternary ammonium compound-resistance protein qacE (P0AGC9); and streptomycin adenyltransferase AadA (AAL08435). In contrast, sequencing of the class 2 integron gene cassettes did not reveal presence of any drug resistance genes.

### qPCR for 16S rRNA gene and integrons

**Figure 2:**
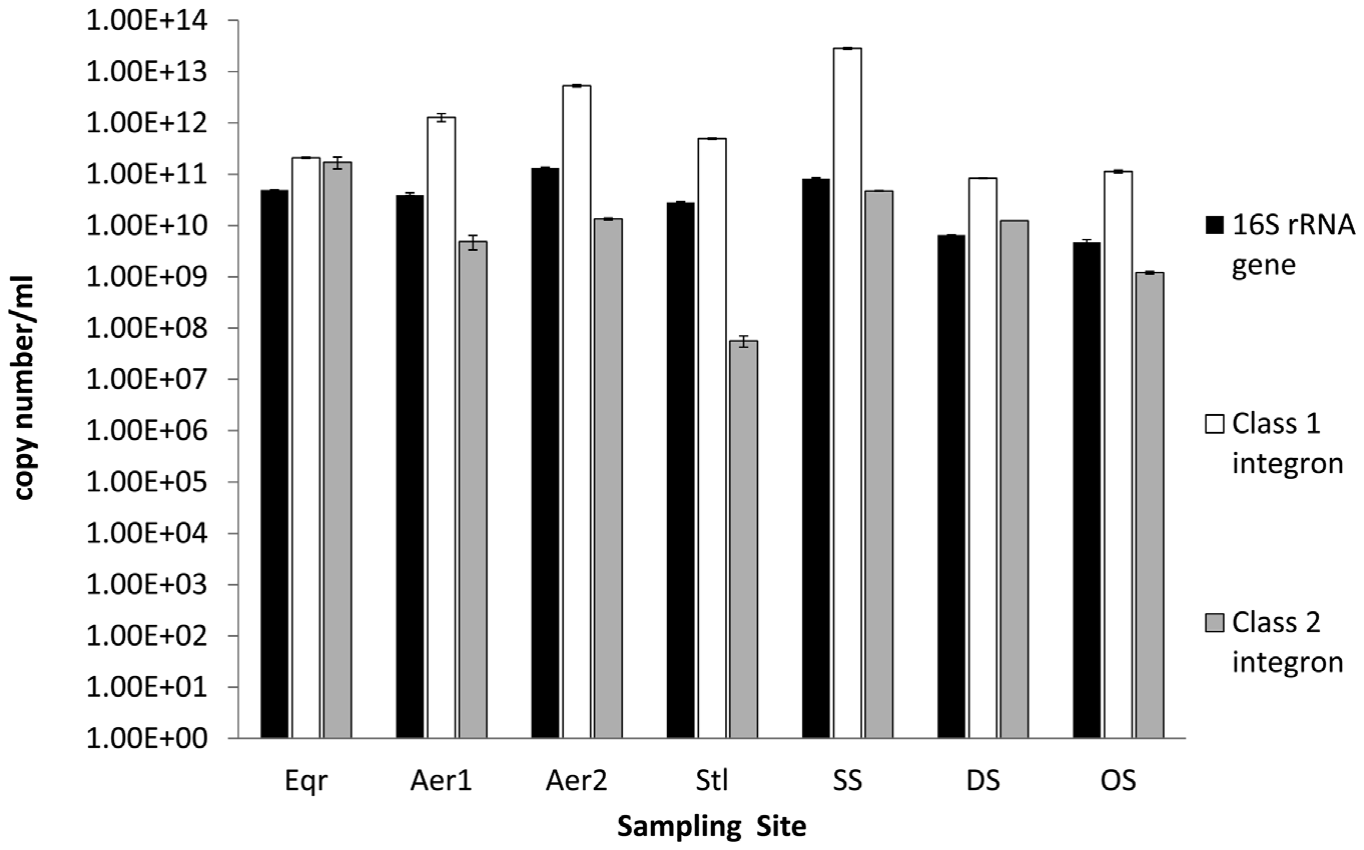
Comparison of bacterial 16S rRNA gene copy number and copy numbers of integron classes 1 and 2. The error bars represent standard deviations of three technical replicates. Abbreviations: Eqr  =  equilibratior; Aer1  =  aeration tank No. 1; Aer2  =  aeration tank No. 2; Stl  =  settling tank; SS  =  secondary sludge; DS  =  dewatered sludge; OS  =  old dried sludge.

The efficiencies of the qPCR were 96, 104 and 109% for 16S rRNA genes, integron classes 1 and 2, respectively. Melting curves obtained for 16S rRNA genes, integron classes 1 and 2 are presented in Figure S1. In the samples from all stages of the treatment process, class 1 integrons were more abundant than class 2 integrons (Figure 2). Class 1 integrons were found at higher levels than 16S rRNA genes in all samples.

## Discussion

High level of multi-drug resistance was observed among the bacterial strains obtained from a WWTP processing collected waste-water from different bulk-drug production facilities. To the best of our knowledge, the levels of multi-drug resistance exhibited by the strains in this study represent the highest reported from any environmental sample [9], [20], [21]. Resistance to more than one antibiotic class was observed in all strains. A great majority (86%) of the strains were resistant to 20 or more antibiotics (Figure 1). Intrinsic resistance in bacteria is well-known, *e.g*., Gram-negative bacteria typically are resistant to vancomycin, as they do not synthesize heavy peptidoglycan cell walls, which is the particular target of vancomycin [37]. Although, some of the isolated strains are expected to be resistant to several antibiotics, intrinsic resistance, alone, does not explain the degree of multi-drug resistance observed in this study. Acquired resistance (or loss of resistance) can be inferred by comparing the antibiotic sensitivity profiles of the different strains of the same species. When comparing all 13 strains identified as *O. intermedium,* there was a difference in the resistance profiles of these strains, one of the *O. intermedium* strain (ER-1) was observed to be sensitive to only one of the 39 tested antibiotics, resistant to 36 and intermediately resistant to 2 antibiotics while, on the other hand, the least resistant strain of *O. intermedium* (A2T-3) demonstrated resistance to 18 antibiotics and intermediate resistance to 7. Such observations suggest that the overall resistance profiles of the strains are, to a large degree, cases of acquired resistance. In a parallel study, genome sequence analysis of the *O. intermedium* strain ER-1 highlighted the underlying mechanisms behind the acquired resistance in this strain [38]. When comparing *O. intermedium* strains with the sensitivity patterns of the reference type strains of *O. intermedium*, the strains in this study were found to be resistant to ciprofloxacin, gentamicin, norfloxacin, chloramphenicol, tetracycline, doxycycline and azithromycin, the antibiotics to which the type strains are sensitive [39]. Similar results were observed when comparing the antibiotic sensitivity patterns of different strains in this study belonging to the same species.

A recent study by Bhullar *et al.,* demonstrated multi-drug resistance in samples from ancient caves, with some strains being resistant to more than 10 antibiotics. They reported very low resistance (less than 2% bacterial strains) to fluoroquinolone antibiotics [9]. D'Costa *et al.,* reported only 10% of the strains from soil to be resistant to ciprofloxacin [40]. This might be attributed to the strictly synthetic nature of fluoroquinolones. In contrast, in this study 62% of the strains were resistant to ciprofloxacin with an additional 20% being intermediately resistant. It seems highly plausible that the high fluoroquinolone selection pressure present in the waste-water of the treatment plant is a driver behind the selection and amplification of such multi-resistant strains. However, it cannot be concluded to what extent these multi-drug resistance genotypes have emerged in this extreme environment as opposed to being enriched from the incoming waste, via the prevailing selection pressure inside the plant. Previous studies addressing the chemical composition of the treated effluent from PETL have found a range of pharmaceuticals at very high levels [16], [17], [23]. Even though fluoroquinolones were the only antibiotic class detected at therapeutic levels in these studies, the exceptional multi-drug resistance patterns in the bacterial strains isolated here extends well beyond quinolone resistance. This could be a result from co-selection and/or that the concentrations of other antibiotics were sufficient to select for the respective resistant strains [15].

Integrons, especially class 1 integrons, are associated with mobile elements commonly containing gene cassettes with drug resistance markers [41]. Integrons were found in 35% of the isolates of genus *Aeromonas* and family *Enterobacteriaceae* from a slaughter-house waste-water treatment plant [42]. The extraordinarily high prevalence of integrons among the strains from the PETL WWTP (95%) suggests a high potential for HGT in this environment. The integron gene cassette of the class 1 integrons showed the presence of aminoglycoside 6′-n-acetyltransferase, aminoglycoside adenyltransferase, OXA-2 beta-lactamase precursor, streptomycin adenyltransferase and dihydrofolate reductase. All of these genes were reported in a metagenomic study of antibiotic-contaminated sediment from the river passing the PETL WWTP [12]. Sequencing of heavier bands than 1.5kb could very well have resulted in detection of a larger variety of resistance genes. Nevertheless, the present study provides evidence that class 1 integrons are associated with resistance genes at this site, data that may be difficult to conclude from metagenomic shotgun sequencing. Kristiansson *et al.,* also showed that the number of class 1 integrons increased down-stream from the PETL WWTP, compared to up-stream [12]. This is in accordance with the higher number of antibiotic resistance genes observed downstream from PETL as well as our finding of a high prevalence of strains with class 1 integrons. No resistance genes were found within class 2 integron gene cassettes. However, since a relatively few 1 kb fragments of gene cassette were sequenced, this is not proof of absence. Class 2 integrons have been shown to be associated with antibiotic resistance genes, although the association of class 2 integrons with antibiotic resistance genes is less widespread than that of class 1 integrons [41], [43]–[45]. This is in accordance with our results.

The qPCR analyses of the total community DNA revealed that the class 1 integrons predominated over the class 2 integrons. The copy number of class 1 integrons was estimated to be higher than that of the 16S rRNA gene in all samples. A bacterium usually contains between 1 and 15 copies of the 16S rRNA gene [46]. The data suggests the occurrence of multiple copies of class 1 integrons in bacteria from the PETL WWTP. Wright *et al.,* reported heavy metal pollution to be associated with an increased abundance of class 1 integrons. The copy numbers of integrons in their study were, however, still 10^2^ to 10^5^ folds lower than the 16S rRNA gene copy number [47]. Gaze *et al.,* estimated that less than 1% of the bacterial cells in textile effluent, contaminated reef, sewage sludge and pig slurry contained class 1 integrons [48]. In contrast, the bacterial communities from within the PETL WWTP possessed, to the best of our knowledge, the highest prevalence of integrons in any contaminated environment reported so far. The ratios of integron classes 1 and 2 varied from approximately similar levels, in the equilibrator, to 10,000 times higher frequencies of the class 1 integrons in samples taken from later stages of the treatment process. The apparent shift might be due to selection of bacteria with class 1 integrons, containing resistance genes, in the later stages of treatment process. Class 2 integrons exhibit lower degree in the diversity of gene cassettes, compared to class 1 integrons, because of the presence of a stop codon in the integrase gene (*int2*) at the 179^th^ position. The stop codon results in formation of a truncated integrase protein which is non-catalytic [41], [49]. However, the described diversity in some class 2 integron gene cassette arrays, is attributed to the ability of class 1 integrases to catalyze the recombination at the attachment site of the class 2 integrons [43]–[45], [50], [51].

The extreme selection pressure in the PETL WWTP is expected to favour antibiotic-resistant bacteria. If many of those resistant bacteria also carry integrons, this, in turn, is expected to lead to a high abundance of integron-carrying bacteria, and ultimately enhance the HGT potential within the entire bacterial community. Another scenario is that the HGT potential itself might be crucial for the survival of bacteria in the PETL WWTP, rather than merely selecting for those bacteria already resistant when entering the plant. It could be the ability of some bacteria to capture resistance genes within the plant, through integron-based capture systems, that have favoured integron-carrying bacteria. The high abundance of integrons in PETL could be a result of both processes acting in concert.

Although no classical human pathogens were isolated among the 93 investigated strains, opportunistic pathogens like *O. intermedium, P. rettgeri,* vancomycin resistant enterococci (VRE) and *Aerococcus* sp., known to cause severe infections under particular conditions, were observed to be highly drug resistant [52]–[62]. These opportunistic pathogens would not be considered to be a direct public health threat. However, if such multi-resistant bacterial strains colonize individuals undergoing antibiotic therapy, these strains are likely to increase dramatically in numbers, increasing the risks of causing infections that are difficult to treat. The other, perhaps larger threat, posed by multi-drug resistant bacterial strains enriched in environments like the PETL WWTP, is the potential for transferring their resistance genes to commensals and, ultimately, to human pathogens. Environmental and human commensal bacteria serve as reservoirs of resistance genes, genes that one after the other have made their way into human pathogens during the past 70 years [4], [5], [63], [64]. Although such events are likely to be rare and difficult to spot, the possibility of this happening in places like the PETL WWTP should not be underestimated.

The PETL WWTP applies an activated sludge technology. This technology, however, results in an unintended but active selection for resistant bacteria as the activated sludge bacteria are exposed to strong antibiotic selection repeatedly during the recycling of the sludge. Additionally, approximately 20% of raw human feces, inevitably containing pathogens, are added daily to maintain biological activity [16]. Furthermore, the treatment plant frequently operates at temperatures above 30°C. Such a close and extended contact among pathogens, resistant bacteria and antibiotics provide an environment with apparent opportunities for transfer of resistance to classical pathogens.

Although it is reasonably clear that propagation and spread of resistant bacterial clones is mainly due to human activity, *i.e*., through misuse and overuse of antibiotics in human and veterinary medicine and agriculture, it is considerably less clear where and under what circumstances resistance factors are assembled into compatible genetic units and further transferred into human pathogens. The extent of multi-drug resistance observed in this study suggests that the kind of selection pressures as found in the PETL WWTP would create theoretically perfect breeding grounds for this to occur. It is not yet known how likely it is that new pathogenic “superbugs” are created in environments like these. However, given the potential threat, the precautionary principle requires proper management of antibiotic manufacture waste to avoid creating such spawning grounds [28].

## Supporting Information

Figure S1 **Melting curves obtained in qPCR analysis.** A) Melting curves for 16S rRNA gene, B) Melting curves for class 1 integrase gene, C) Melting curves for class 2 integrase gene.
(TIF)

Table S1 **The cut off values of zones of inhibition used for determination of resistant, intermediately resistant or sensitive nature of the isolated strains.**
(DOC)

Table S2 **Prevalence of class 1 and class 2 integrons among the strains from the PETL WWTP.**
(DOC)

File S1 **Antibiotic sensitivity data for the individual strains.**
(XLSX)
