# Supplementary figures and images for: A Treatment Plant Receiving Waste Water from Multiple Bulk Drug Manufacturers Is a Reservoir for Highly Multi-Drug Resistant Integron-Bearing Bacteria
Source: PLoS One. 2013 Oct 29;8(10):e77310. doi: 10.1371/journal.pone.0077310 (PMC3812170; doi:10.1371/journal.pone.0077310)

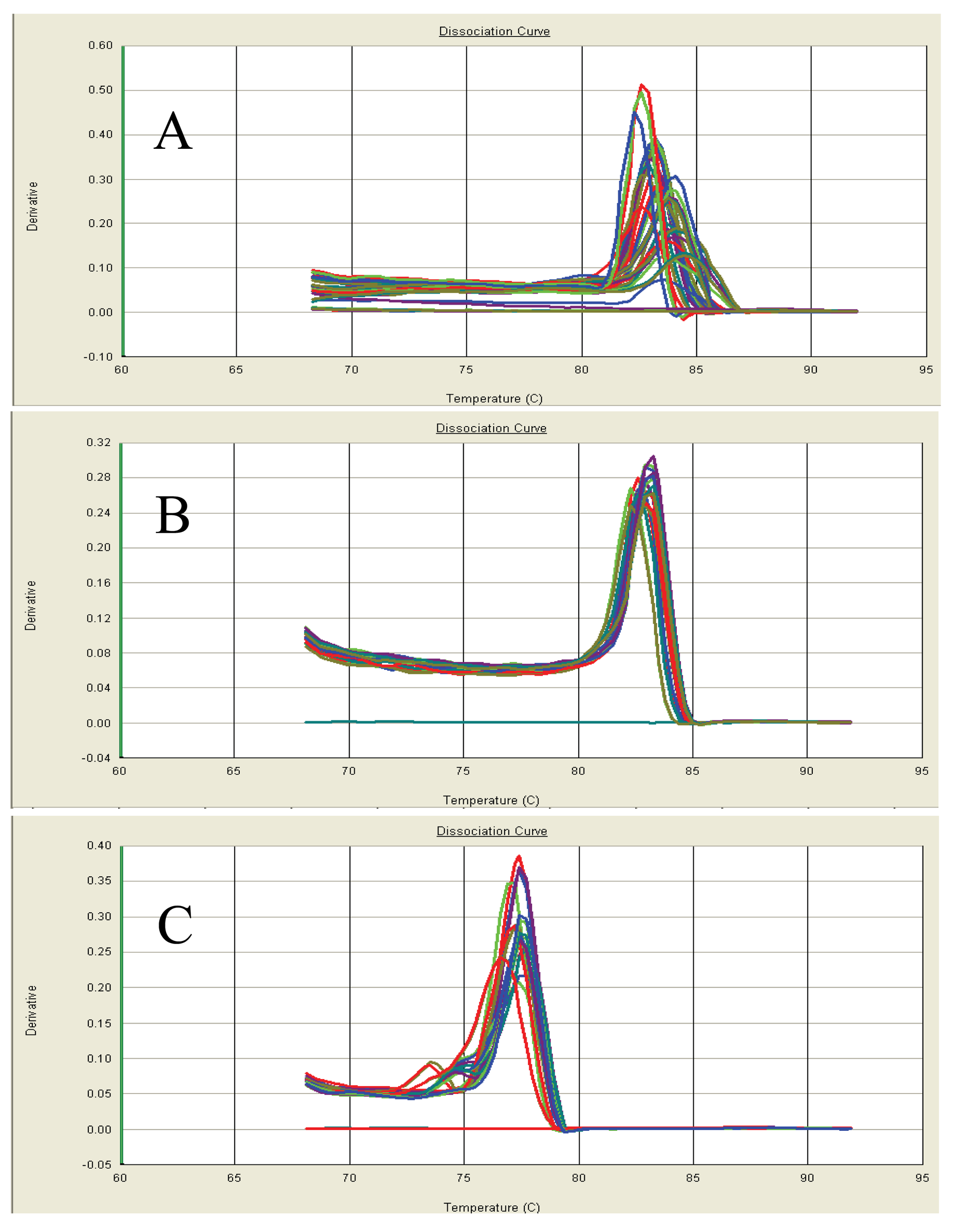

Supplement: Figure S1 — Melting curves obtained in qPCR analysis. A) Melting curves for 16S rRNA gene, B) Melting curves for class 1 integrase gene, C) Melting curves for class 2 integrase gene. (TIF) [file pone.0077310.s001.tif]
