# Supplementary material for: A Treatment Plant Receiving Waste Water from Multiple Bulk Drug Manufacturers Is a Reservoir for Highly Multi-Drug Resistant Integron-Bearing Bacteria
Source: PLoS One. 2013 Oct 29;8(10):e77310. doi: 10.1371/journal.pone.0077310 (PMC3812170; doi:10.1371/journal.pone.0077310)
Supplement: Table S1 — The cut off values of zones of inhibition used for determination of resistant, intermediately resistant or sensitive nature of the isolated strains. (DOC) [file pone.0077310.s002.doc]

**Supplementary table S1**: The cut off values of zone of inhibition used for determination of resistance, intermediate resistance or sensitive nature of the strains.

|  | **Antibiotic** | Qty per disc(µg) | Resistant | Intermediate | Sensitive |
| --- | --- | --- | --- | --- | --- |
|  |  |  | zone of inhibition in mm | | |
| 1 | Gentamicin | 10 | ≤ 12 | 13-14 | ≥ 15 |
| 2 | Tobramycin | 10 | ≤ 12 | 13-14 | ≥ 15 |
| 3 | Streptomycin | 10 | ≤ 11 | 12-14 | ≥ 15 |
| 4 | Cephalexin(I) | 30 | ≤ 14 | 15-17 | ≥ 18 |
| 5 | Cephalothin(I) | 30 | ≤ 14 | 15-17 | ≥ 18 |
| 6 | Cephoxitin(II) | 30 | ≤ 14 | 15-17 | ≥ 18 |
| 7 | Cefaclor(II) | 30 | ≤1 4 | 15-17 | ≥ 18 |
| 8 | Ceftazidime(III) | 30 | ≤ 14 | 15-17 | ≥ 18 |
| 9 | Ceftriaxone(III) | 30 | ≤ 13 | 14-20 | ≥ 21 |
| 10 | Cephotaxime(III) | 30 | ≤ 14 | 15-22 | ≥ 23 |
| 11 | Teicoplanin | 30 | ≤ 10 | 11-13 | ≥ 14 |
| 12 | Vancomycin | 30 | ≤ 14 | --- | ≥ 15 |
| 13 | Azithromycin | 15 | ≤ 13 | 14-17 | ≥ 18 |
| 14 | Erythromicin | 15 | ≤ 13 | 14-17 | ≥ 18 |
| 15 | Aztreonam | 30 | ≤ 15 | 16-21 | ≥ 22 |
| 16 | Ampicillin | 10 | ≤ 13 | 14-16 | ≥ 17 |
| 17 | Augmentin | 30 | ≤ 13 | 14-17 | ≥ 18 |
| 18 | Methicillin | 5 | ≤ 9 | 10-13 | ≥ 14 |
| 19 | Nalidixic acid | 30 | ≤ 13 | 14-18 | ≥ 19 |
| 20 | Penicillin-G | 10 Unit | ≤ 19 | 20-27 | ≥ 28 |
| 21 | Piperacillin/Tazobactum | 100/10 | ≤ 17 | 18-20 | ≥ 21 |
| 22 | Ticarcillin | 75 | ≤ 14 | 15-19 | ≥ 20 |
| 23 | Mecillinam | 10 | ≤ 11 | 12-14 | ≥ 15 |
| 24 | Colistin | 25 | ≤ 10 | --- | ≥ 11 |
| 25 | Ciprofloxacin | 5 | ≤ 15 | 16-20 | ≥ 21 |
| 26 | Gatifloxacin | 30 | ≤ 14 | 15-17 | ≥ 18 |
| 27 | Norfloxacin | 10 | ≤ 12 | 13-16 | ≥ 17 |
| 28 | Ofloxacin | 5 | ≤ 12 | 13-15 | ≥ 16 |
| 29 | Sparfloxacin | 5 | ≤ 15 | 16-18 | ≥ 19 |
| 30 | Sulfamethoxazole | 25 | ≤ 12 | 13-16 | ≥ 17 |
| 31 | Co-Trimoxazole | 25 | ≤ 10 | 11-15 | ≥ 16 |
| 32 | Trimethoprim | 5 | ≤ 10 | 11-15 | ≥ 16 |
| 33 | Doxycycline HCl | 30 | ≤ 12 | 13-15 | ≥ 16 |
| 34 | Netillin | 30 | ≤ 12 | 13-14 | ≥ 15 |
| 35 | Tetracycline | 50 | ≤ 14 | 15-18 | ≥ 19 |
| 36 | Chloramphenicol | 25 | ≤ 12 | 13-17 | ≥ 18 |
| 37 | Fusidic acid | 10 | ≤ 19 | 20-21 | ≥ 22 |
| 38 | Nitrofurantoin | 300 | ≤ 14 | 15-16 | ≥ 17 |
| 39 | Novobiocin | 5 | ≤ 17 | 18-21 | ≥ 22 |

Legend: ≤ = less than or equal to; ≥ = more than or equal to.
