# Supplementary material for: A Treatment Plant Receiving Waste Water from Multiple Bulk Drug Manufacturers Is a Reservoir for Highly Multi-Drug Resistant Integron-Bearing Bacteria
Source: PLoS One. 2013 Oct 29;8(10):e77310. doi: 10.1371/journal.pone.0077310 (PMC3812170; doi:10.1371/journal.pone.0077310)
Supplement: Table S2 — Prevalence of class 1 and class 2 integrons among the strains from the PETL WWTP. (DOC) [file pone.0077310.s003.doc]

Supplementary table2: Prevalence of class1 and class2 integrons among the strains from PETL WWTP.

| **isolate** | **identity** | **Class 1 integron** | **Class 2integron** |
| --- | --- | --- | --- |
| SR-1 | *Staphylococcus cohnii* | - | + |
| SR-3 | *Enterobacter hormaechei* | + | + |
| SR-4 | *Pseudomonas plecoglossicida* | + | + |
| SR-5 | *Citrobacter freundii* | + | + |
| SR-6 | *Bordetella trematum* | - | + |
| SR-7 | *Pseudomonas plecoglossicida* | + | + |
| SR-8 | *Pseudomonas plecoglossicida* | + | + |
| SR-9 | *Aerococcus urinaeequi* | + | + |
| SR-11 | *Pseudomonas fulva* | + | + |
| SR-12 | *Ochrobactrum intermedium* | + | - |
| SR-13 | *Bordetella trematum* | + | - |
| SR-14 | *Pseudomonas anguilliseptica* | + | + |
| ST-1 | *Alcaligenes faecalis* | + | + |
| ST-2 | *Alcaligenes faecalis* | + | + |
| ST-3 | *Pseudomonas sp* | + | + |
| ST-4 | *Pseudomonas sp* | + | + |
| ST-5 | *Rheinheimera aquimaris* | + | + |
| ST-6 | *Castellaniella denitrificans* | + | + |
| ST-7 | *Brevundimonas naejangsanensis* | + | + |
| ST-8 | *Castellaniella denitrificans* | + | + |
| A1R-2 | *Ochrobactrum intermedium* | + | + |
| A1R-4 | *Bacillus flexus* | - | - |
| A1R-6 | *Ochrobactrum oryzae* | + | - |
| A1R-7 | *Ochrobactrum intermedium* | + | + |
| A1R-9 | *Ochrobactrum intermedium* | + | + |
| A1R-10 | *Advenella mimigardefordensis* | - | + |
| A1T-2 | *Pseudomonas stutzeri* | + | + |
| A1T-3 | *Pseudomonas stutzeri* | + | + |
| A1T-4 | *Ochrobactrum intermedium* | + | + |
| A1T-5 | *Ochrobactrum intermedium* | + | + |
| A1T-7 | *Enterococcus casseliflavus* | + | + |
| A1T-8 | *Bacillus cereus* | + | + |
| A2R-4 | *Ochrobactrum intermedium* | + | + |
| A2R-5 | *Bacillus safensis* | - | + |
| A2R-6 | *Bacillus subtilis* | - | + |
| A2R-8 | *Ochrobactrum intermedium* | + | + |
| A2T-3 | *Ochrobactrum intermedium* | + | - |
| A2T-4 | *Bacillus cereus* | + | + |
| A2T-5 | *Advenella mimigardefordensis* | + | + |
| A2T-6 | *Pseudomonas caeni* | + | + |
| A2T-8 | *Aquamicrobium defluvii* | - | + |
| ER-1 | *Ochrobactrum intermedium* | + | - |
| ER-2 | *Ochrobactrum intermedium* | + | + |
| ER-4 | *Pseudomonas stutzeri* | + | + |
| ER-5 | *Bacillus cereus* | + | + |
| ET-1 | *Pseudomonas caeni* | + | + |
| ET-2 | *Pseudomonas peli* | + | + |
| ET-4 | *Bacillus cereus* | + | + |
| ET-5 | *Pseudomonas caeni* | + | + |
| ET-6 | *Pseudomonas caeni* | - | + |
| ET-7 | *Alcaligenes sp.* | - | + |
| DSR-1 | *Bacillus cereus* | - | - |
| DSR-2 | *Bacillus cereus* | - | + |
| DSR-4 | *Providencia rettgeri* | + | + |
| DSR-5 | *Providencia rettgeri* | + | + |
| DSR-6 | *Aerococcus urinaeequi* | + | + |
| DST-1 | *Pseudomonas caeni* | + | + |
| DST-2 | *Pseudomonas caeni* | + | - |
| DST-3 | *Pseudomonas caeni* | + | - |
| DST-4 | *Pseudomonas caeni* | + | + |
| DST-5 | *Pseudomonas caeni* | + | + |
| DST-6 | *Brevundimonas diminuta* | + | + |
| DST-7 | *Pseudomonas caeni* | - | + |
| DST-8 | *Pseudomonas caeni* | + | + |
| OSR-1 | *Bacillus subtilis* | - | - |
| OSR-3 | *Providencia rettgeri* | + | + |
| OSR-4 | *Pseudomonas xanthomarina* | + | + |
| OST-1 | *Aerococcus urinaeequi* | + | + |
| OST-2 | *Alcaligenes faecalis* | - | + |
| OST-3 | *Brevibacterium samyangense* | - | + |
| OST-4 | *Corynebacterium sp.* | - | + |
| OST-5 | *Enterococcus italicus* | + | + |
| OST-6 | *Enterococcus gallinarum* | + | + |
| OST-9 | *Alcaligenes faecalis* | + | - |
| OST-10 | *Enterococcus italicus* | + | + |
| OST-11 | *Pseudomonas stutzeri* | + | - |
| OST-12 | *Aerococcus viridans* | + | + |
| OST-13 | *Paenalcaligenes sp.* | + | - |
| OST-14 | *Alcaligenes faecalis* | + | - |
| SSR-1 | *Bacillus subtilis* | - | - |
| SSR-2 | *Bacillus subtilis* | - | - |
| SSR-3 | *Bacillus thuringiensis* | + | - |
| SSR-4 | *Providencia rettgeri* | + | + |
| SSR-5 | *Providencia rettgeri* | + | + |
| SSR-6 | *Ochrobactrum intermedium* | + | - |
| SST-1 | *Pseudomonas caeni* | + | + |
| SST-2 | *Pseudomonas caeni* | - | + |
| SST-3 | *Pseudomonas caeni* | + | + |
| SST-4 | *Pseudomonas caeni* | + | + |
| SST-5 | *Pseudomonas caeni* | + | + |
| SST-6 | *Pseudomonas caeni* | + | + |
| SST-7 | *Ochrobactrum intermedium* | + | - |
| SST-8 | *Pseudomonas caeni* | + | + |

Legend: + = Presence of integron, - = Absence of integron.

Source of strains: Strains ER1 to ER-5 and ET-1 to ET-7 were isolated from the equilibratior tank; A1R-2 to A1R-10 and A1T2 to A1T8 were isolated from aeration tank No. 1; A2R4 to A2R-8 and A2T-3 to A2T-8 were isolated from aeration tank No. 2; SR-1 to SR-14 and ST-1 to ST-8 were isolated from the settling tank; SSR-1 to SSR-6 and SST-1 to SST-8 were isolated from secondary sludge; DSR-1 to DSR-6 and DST-1 to DST-8 were isolated from dewatered sludge; OSR-1 to OSR-4 and OST-1 to OST-14 were isolated from old dried sludge.
